# The Cholera

**Published:** 1891-05-09

**Authors:** 


### THE PRACTITIONER'S MIRROR AND RETROSPECT.

*[The Editor will be glad to receive offers of co-operation and contributions from members of the profession. All letters should be addressed to* THE EDITOR, THE LODGE, PORCHESTER SQUARE, LONDON, W.]

### MEDICINE.

### CHOLERA.

The investigations of Dr. D. D. Cunningham in reference to the bacteriology of cholera cannot fail to throw light on some of the difficulties in determining which particular microbe should be regarded as the immediate cause of the disease. He has studied the life history of the comma bacillus not only in pure cultivations, that is, under artificial conditions, but also in its natural habitat of foul water and soil, and in the presence of the numerous other organisms with which these normally swarm. Under such conditions he found that so long as these retained their natural condition, the commas were singularly incapable of maintaining their existence, and tended to disappear very rapidly, various straight bacilli and micrococci being found after a time, but no commas. In fairly clean water they disappeared in four or five days, and in foul water in from five to nine days. In ordinary garden soil they were able to maintain their existence for ten to twenty-six days, but when this was mixed with healthy fœces they were absent after six to nine days. The strongly acid fermentation which accompanies fæcal decomposition was very fatal to the commas. When, however, these media were partially sterilised by boiling, and large numbers of the schizomycetes naturally present were killed in this way, the comma bacilli were able to live for twenty-five days in the case of foul water, and they were still present in forty-seven days in a mixture of earth and fæces. Professor Sirena, of Palermo, has been able to confirm these observations of Dr. Cunningham. His researches showed that in drinking or spring water the comma bacillus dies in from five to eight days, and in well water in from twenty-four hours to six days. In sea water it lives on an average till the fourth day, while in sea water rendered impure by the admixture of sewage, it survives only from twenty-four to forty-eight hours. Its duration in water or liquid or moist substances seems to depend on the presence of saprophytes or other antagonistic microbes found there, for the more abundantly these occur, the more rapidly do the commas disappear. It has similarly been shown by Dr. Heine that the commas are destroyed in milk on its undergoing acid fermentation, and that the quicker milk turns sour the sooner are the cholera germs killed. Although these researches are of value from a prophylactic point of view, methods of treatment based on the alleged germicidal properties of the drugs employed have not been attended with much success. An article on "Cholera as a Neurosis," by Dr. Hackin, in the *Medical Age*, draws attention to his treatment of this disease. Dr. Hackin believes that practical medicine has suffered much from the invasion of new theories as well as experimental methods. He observes that traditional pathology has given way to experimental pathology, and the spontaneous maladies to those which may be artificially provoked upon the inferior animals. Etiology has assuredly been much simplified by the discovery of the microbe, but it is very doubtful whether scientific medicine has profited much by these methods; observation has been diverted from the right path of clinical observation, and therapeutic experience by the fallacious charms of the germ theory. In most instances it seems to have caused physicians to forget their mission, to have prevented the relief of the sick, and to have produced misconceptions of the disease. Time has only confirmed the words of Trichum, when, apropos of the cholera bacillus, he adjured the members present, despite the murmurs of some, not to believe that this discovery definitely settled the question, any more than the knowledge of the bacillus of tuberculosis would exterminate pulmonary phthisis. However, be the origin of cholera as it may—either miasmatic or bacillary, telluric or meteorological—its neurotic character is most clear. Mr. Sedgwick attributes all the phenomena of cholera to the troubles produced in the functions of the centres of the sympathetic system. Delpech has found traces of inflammation of the semilunar ganglia in patients who had succumbed to cholera. Dr. Johnson admits that the specific poison acts first on the blood or the intestinal tube, then on certain parts of the nervous system, particularly on the sympathetic and the nervous centres. The influence of the nervous system is accordingly very manifest even in the predominant symptoms, whether subjective or objective. The vomitings, and the numerous stools, evidently are connected with the attack upon the nervous apparatus of the stomach, and of the intestines; the choleraic crisis, the cramps, the vertigo, the anxiety, the spasms, the tremblings, have a nervous origin. It is to the vasomotors, then, that we must refer the depression of the functions of respiration and circulation, the gravest symptoms of cholera. Proofs of this nervous origin may be found in the rapid deaths due to dry cholera, which are to be observed during certain epidemics, and in which the patients had enjoyed apparently good health; the fatal result being preventible by the use of hydrocyanic acid or the dilute virus of *Upas antiar*. The prompt cures which we may observe in cases, apparently the most desperate, conflict with the opinion that we have to deal with serious organic affections. Dr. Hackin, after referring to the researches of Claude Bernard, which threw a new light on the pathology of cholera, goes on to say that it seems to him that we shall find in the antagonism of the pneumogastric nerves the means of checking the action of the sympathetic nerves in cholera. In the absence of an epidemic of true cholera, he was forced to test the theory practically in cases of English cholera, or cholerina, and in every case he observed that by stimulating the pneumogastric in the neck, or by developing its inhibitory power, the vomiting, the stools, and the cramps were arrested. But when, in 1887, a very severe epidemic of cholera broke out at Malta, this mode of treatment was employed with a superiority of results as evident as in the less grave epidemic in which he had used it in England. As the author remarks, the simplicity of the treatment is a high recommendation. In all attacks of cholera, whatever the stage, it is sufficient to apply with a brush the epispastic liquor of the British pharmacopœia on the branches of the pneumogastric in the neck, on the mastoids under the eye, covering three inches of surface. The effect is generally instantaneous, the stools, the cramps, and the vomiting cease; the pulse regains its power, and the warmth returns; the patient falls asleep, and all the morbid phenomena are annihilated before the vesication is to be seen. In the crisis of cholera these applications to the vagus seem to paralyse completely the sympathetic nerve in the stomach or the intestines. In general it is preferable to produce the vesication on the right side, Coleman having demonstrated that the right pneumogastric commands the slender intestine, but if necessary the left vagus can be put under contribution. The vagus is an inhibitory nerve, and possesses an action antagonistic to that of the sympathetic nerve on the heart.

By stimulating the sympathetic part of the heart its contractions are augmented, but by acting thus on the vagus we can determine the arrest of the heart in full diastole. The stimulation of the vagus gives rise to an important indication; that is, the re-establishment of the cardio-inhibitory functions of this nerve, which are evidently absent in cholera. The violent contractions and the palpitation of the heart cease; the energy of its cavities, especially those of the left side, returns; the congestion of the pulmonary and cutaneous system disappears. Simon and others have shown that in cholera the left side of the heart is generally empty, whilst the right side is distended and filled with blood. From these and various other physiological details, continues Dr. Hackin, we may readily deduce the mode of treatment which he has proposed. The practical application of admitted physiological and pathological principles, and the discovery of constant relatives of cause and effect, suggest the idea of a well-defined law in this affection. The author concludes by citing examples of the action of his mode of treatment in the three principal divisions of cholera.

1. Cholera infantum.—This was in a child six years old, first seen at 11.30 a.m. He found the child enfeebled, cold; had vomited and had repeated passages. Had been seized at 6 a.m. that morning, and had vomited at least every quarter of an hour up to the time of his arrival. No medicine was prescribed, but the vesicant liquid was applied under both eyes and to the neck. In half an hour the child was asleep and it slept all night. The next morning the child had practically recovered, having had no more vomiting and no more diarrhœa.

2. Cholera nostras.—Patient a policeman.—On arrival of the doctor he presented the following symptoms: Vomiting, feeble pulse, violent cramp, palpitation of the heart, great debility, coldness of the extremities, numerous rice-like stools. He told me that on arriving at the police barracks, he had been seized with profuse vomiting, then, after the lapse of a half-hour, with numerous stools and cramps. The attacks succeeded each other every quarter-hour. No medicine was given but the vesicant applied under both eyes. At 10.30 he was convalescing and had no more passages or attacks of vomiting.

3. Cholera Asiatic. — Case related by Dr. Tuglott, Malta, of a boatman, age 42. His wife died of spasmodic cholera and he had been waiting on her, and one hour after her death he was seized with intense diarrhœa, rice-like motions, eyes glaucous, lips violet, body cold, voice feeble, pulse slender, respiration shallow, thirst intense, painful cramp in the lower extremities, general debility, and suppression of urine. Was treated by injections of ether—profound vesication applied to the vagus, the two sides and of the neck. Two hours later the patient had slept nearly an hour. The cramps had ceased, the pulse had regained its vigour, and the urine was passed freely. In the afternoon the patient was feeble but in full convalescence.

Such results are indeed most encouraging and afford strong clinical evidence of the truth of Dr. Hackin's views, and we trust that the results of a more extended trial may confirm the efficacy of this method.

